# Effects of Smoking on Diabetic Nephropathy

**DOI:** 10.3389/fcdhc.2022.826383

**Published:** 2022-02-23

**Authors:** Yasemin Gündoğdu, İnan Anaforoğlu

**Affiliations:** ^1^ School of Medicine, Department of Internal Medicine, Acıbadem Mehmet Ali Aydınlar University, Istanbul, Turkey; ^2^ School of Medicine, Department of Endocrinology and Metabolism, Acıbadem Mehmet Ali Aydınlar University, Istanbul, Turkey

**Keywords:** tobacco, smoking, diabetes, diabetic nephropathy, diabetic kidney disease

## Abstract

Diabetes is a systemic metabolic disease with serious complications that cause significant stress on the healthcare system. Diabetic kidney disease is the primary cause of end stage renal disease globally and its progression is accelerated by various factors. Another major healthcare hazard is tobacco consumption and smoking has deleterious effects on renal physiology. Prominent factors are defined as sympathetic activity, atherosclerosis, oxidative stress and dyslipidemia. This review aims to enlighten the mechanism underlying the cumulative negative effect of simultaneous exposure to hyperglycemia and nicotine.

## Introduction

The diabetes mellitus (DM) pandemic has risen rapidly over the last decades making the global burden of DM a substantial cause of stress on the healthcare system. The global prevalence of DM was 8.4% in 2017 while it is estimated that this number will rise up to 9.9% in 2045 (1). According to the WHO, DM is the ninth cause of mortality and 48% of the patients with DM die before the age of 70 (2). In 2018, 34.5% of all US adults had prediabetes, the condition that has a high risk of progression to type 2 diabetes mellitus(T2DM) (3). Younger people are being diagnosed with DM today and this causes an increase in the incidence of diabetic complications. A significant complication of long term hyperglycemia is diabetic nephropathy and it is the leading cause of kidney failure in the world (4).

Although genetic predisposition has been shown, diabetic kidney disease (DKD) is a progressive, multifactorial condition with preventable environmental risk factors involved (5). It manifests as progressive albuminuria, increased blood pressure and decreased glomerular filtration rate (GFR) (6). Uncontrolled hypertension, poor glycemic control, smoking, alcoholism, obesity, hyperlipidemia, low physical activity are among the major modifiable risk factors. Age, male sex, duration of DM and family history of DKD are among the major non-modifiable risk factors. It is important to identify risk factors for DKD in patients in order to take preventative actions accordingly.

Cigarette smoking is the primary preventable cause of death and disability. Reported by the center of disease control (CDC), The United States spent over 300 billion dollars last year due to tobacco related healthcare (7). With the rise of new regulations regarding marihuana legalization and various novel alternative to cigarettes, global tobacco consumption is also increasing.

Smoking was reported as a risk factor for DKD, independent from well-documented risk factors like hypertension, glycemic control and age progression as shown in a meta-analysis by Llao et al. (8). Smoking accelerates DKD progression in both Type 1 DM and Type 2 DM (9). According to CDC, 21.8% of patients with DM were tobacco users in 2018 (10).

Early microscopic manifestations of DKD can be listed as mesangial matrix deposition, thickened glomerular basement membranes(GBM). Pedicels, the foot processes of podocytes, are widened and this results in impaired filtration slits (11).

The precise mechanism of smoking mediated DKD progression is not clearly documented. The major triggers are noted as, sustained sympathetic over activity, atherosclerosis, oxidative stress and hyperlipidemia (12). These factors are closely related to each other since DM is a multisystemic metabolic disease.

Therefore, it is especially important to comprehend the mechanisms underlying the positive impact of smoking cessation on diabetic patients, that already is a major strain on the healthcare system. In this mini-review, we aim to report the complex interplay between smoking and DM while emphasizing the additive effect of smoking on DKD progression, that causes significant morbidity and mortality.

## Sustained Sympathetic Overactivity

In patient with DM who smoke, a metabolic profile is generated where usually hypertension co-exists. This issue reflects to clinical practice for smoking cessation, optimal glycemic control and maintaining target blood pressure are the goals to prevent diabetic complications.

**Figure 1:**
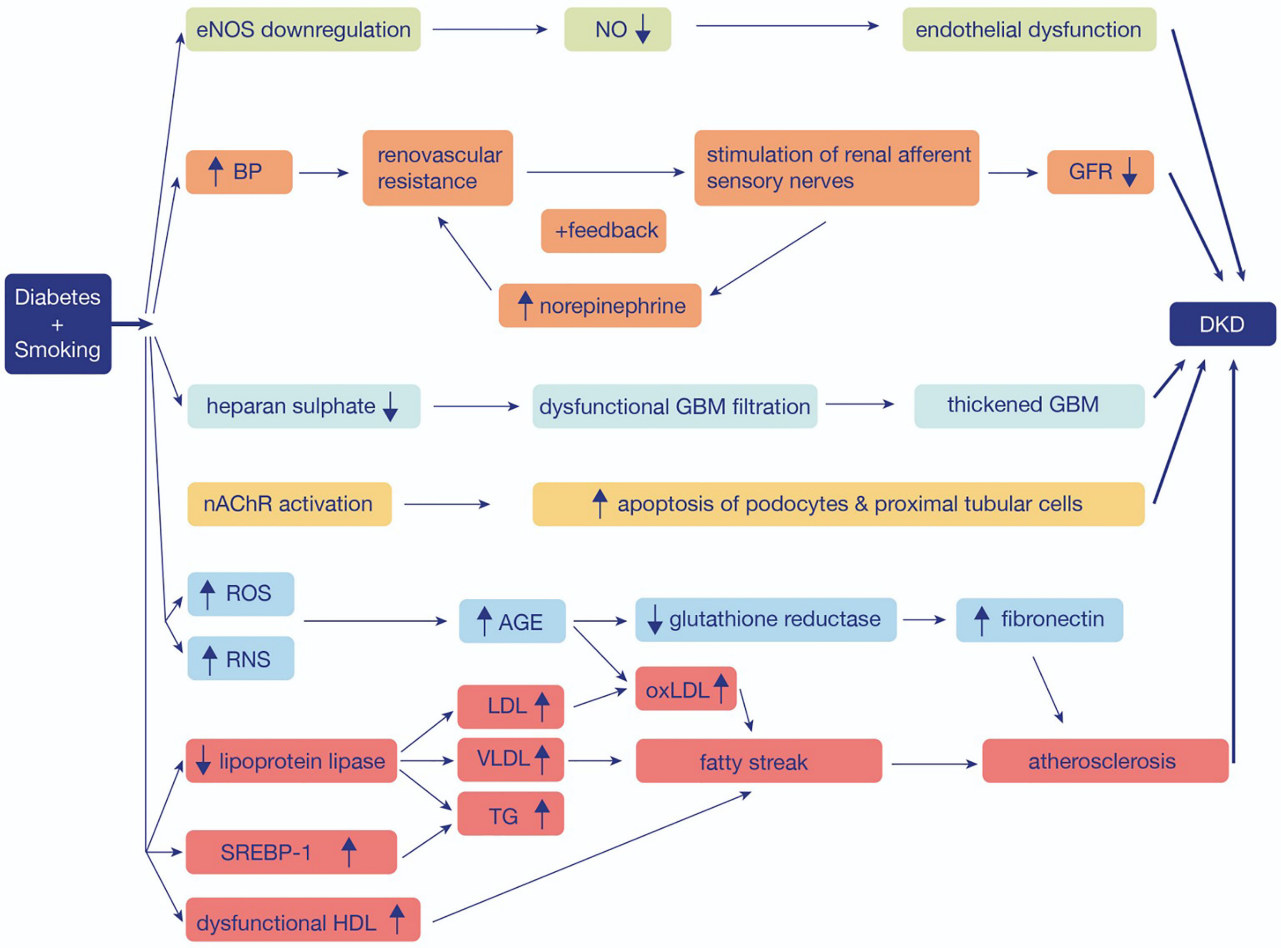
The mechanistic pathway of the role of smoking and diabetes in the progression of diabetic kidney disease. eNOS, Endothelial nitric oxide synthase; NO, Nitric oxide; BP, Blood pressure; GFR, Glomerular Filtration Rate; DKD, Diabetic Kidney Disease; GBM, Glomerular basement membrane; nAChR, Nicotinic acetylcholine receptor; ROS, Reactive oxygen species; RNS, Reactive nitrogen species; AGE, Advanced glycation end products; LDL, Low-density lipoprotein; VLDL, Very Low Density Lipoprotein; TG, Triglyceride; oxLDL, oxidized Low-density lipoprotein; SREBP-1, sterol regulatory element binding protein-1; HDL, High-density lipoprotein.

In healthy individuals, vasoconstriction and vasodilation form a homeostatic balance. Smoking and chronic hyperglycemia result in downregulation of endothelial nitric oxide synthase (eNOS), the enzyme that produces NO (**Figure 1**) (12). Decreased NO levels lead to reduced endothelial vasodilation and cause endothelial dysfunction, which is the hallmark of DKD (13). In rats exposed to tobacco smoke, the intravascular nitrite/nitrate concentration decreased which marks the impairment of Ach dependent endothelial vasodilation (14). Insulin directly induces vasodilation and activates NO production from the endothelium.

It was shown in human endothelial cells in hyperglycemic medium that insulin-stimulated NO production was significantly reduced (15). The regulatory effect of insulin on the vasomotor tone was investigated in various studies and the production of endothelin-1, a powerful vasoconstrictor was proposed as the major mechanism of vasoconstriction in insulin resistance (16).

In patients with type 1diabetes mellitus (T1DM) with absolute insulin deficiency and in patients with T2DM with relative insulin deficiency forms a setting for endothelial dysfunction (11).

Other than decreased eNOS activity, the other cause of NO depletion associated with endothelial dysfunction in DM and smoking is its use in the oxidative pathway (17). Despite the renoprotective effect of NO in physiological states, NO may react with superoxide, the major oxidant molecule, and form peroxynitrate in inflammatory states (18). Peroxynitrate is the chief reactive nitrogen species (RNS) which over activates poly ADP-Ribose polymerase (PARP) mediated consumption of NAD (19). Although the main role of PARP is the maintenance of genome integrity, intense stimulation by oxidative stress leads to harmful levels of ATP, resulting in apoptosis (20).

Renal blood flow is dependent on multiple factors which are organized by neurohumoral feedback loop. This phenomenon differentiates the kidney from the rest of the body. Oxygen demand is not the primary factor affecting renal blood flow (21). It has been clearly shown that smoking leads to high blood pressure that results in increased renovascular resistance and decreased glomerular filtration rate. The cumulative effect of DM and smoking lead to sympathetic over activity and decreased GFR that eventually leads to end-stage renal disease (22). Oxidative stress, ischemia and chronic hyperglycemia overstimulate the renal afferent sensory nerves projecting to central pathways like the rostral ventrolateral medulla (RVLM) and eventually cause norepinephrine secretion that exacerbates DKD progression by causing a sustained positive feedback loop (22).

## Atherosclerosis

Phenotypic alterations of pericytes, low-density lipoprotein (LDL) molecular modifications and formation of fatty streaks are the initial manifestations of DM that lead to atherosclerosis (23). Oxidative stress and formation of Advanced glycation end products (AGE) then further accelerate the development of atherosclerosis.

In a study by Lhotta et al. where 107 renal pathology specimens were analyzed, myointimal hyperplasia of small arteries was found to be twice as frequent in smokers when compared with non-smokers (24). Similar to these findings, Baggio et al. demonstrated that smokers had thicker GBM when compared to non-smokers (461 vs. 398nm, respectively) (6). Arteriolar hyalinosis and thickened GBM provoke a mechanical scaffold for increase in GFR and consequent proteinuria. Various biochemical alterations occur at the GBM of smokers that change the permeability of the membrane. Albuminuria is accelerated as the glycosaminoglycan metabolism is impaired, the charge density of the GBM shifts due to decreased heparin sulfate (6). Reduction in heparin sulfate contributes to the dysfunctional filtration membrane in patients with DM.

Nicotine activates the nicotinic acetylcholine receptors (nAChRs) on the mesangial cells lead to mesangial cell proliferation. Nicotine stimulates apoptosis of proximal tubular cells which also contain nAChRs. The nAChRs on podocytes however, are a novel discovery which illuminated the pathogenesis of podocytopathy mediated proteinuria in DKD (25). It was shown that cleaved caspase-3 and Bax, pro-apoptotoic molecules, expression was increased in rats exposed to tobacco smoke (25). Smoking associated podocyte apoptosis is an important emerging area of investigation to understand the mechanism of proteinuria in DKD (21). Indirectly, nicotinamide adenine dinucleotide phosphate (NADPH) is activated in smokers and this results in (ROS) generation. These findings were exhibited in rats exposed to nicotine had elevated pro-apoptotic enzymes due to excess ROS formation (25). Nicotine decreased nephrin expression on podocytes, marking prominent podocyte cell injury.

Proteins and lipids that are exposed to autooxidated glucose become glycated and these AGE accumulate in the vascular endothelium. Chronic hyperglycemia mediated AGE accumulation is one of the key factors contributing to the higher rate of cardiovascular events encountered in patients with DM. Tobacco itself and smoking have been shown to induce AGE formation and increase its deposition in tissues (26). AGE are proinflammatory substances that lead to activation of macrophages and cytokine formation which have an important role in the pathogenesis of the atherosclerotic plaque (23). Endothelial cells internalize excess AGE by phagocytosis and this contributes to microvascular damage mainly demonstrated at renal and retinal vessels (23).

Another contributing factor to atherosclerosis in patients with DM who smoke is dysfunctional HDL levels (27). The arteriolar macrophages that take up modified LDL molecules make up foam cells that are the predominant cells in fatty streaks (28). The cholesterol efflux from foam cells is conducted by HDL *via* molecules like ATP-binding cassette transporters (29). The conveying of cholesterol from periphery to the liver termed as reverse cholesterol transport is the mechanism of how HDL prevents and reduces atherosclerosis (27). It is an established concept that patients with DM and smokers have reduced HDL levels (30, 31). Notwithstanding lower HDL levels, various conformational changes result in dysfunctional HDL molecules with diminished capability of atheroprotection (27). A study on a cohort with DKD revealed that dysfunctional HDL molecules decreased protective NO levels and interfered with endothelial repairing systems (32). The levels of Apolipoprotein A-1, the major component of HDL particles, are decreased in smokers and this proceeds to dysfunctional HDL biosynthesis (30).

## Oxidative Stress

Oxidative stress occurs when the antioxidant pathways can no longer counterbalance ROS formation rate. The expression of Transforming growth factor (TGF-B), which is a prominent fibrogenic and sclerotic cytokine is increased due to oxidative stress induced by hyperglycemic states (33). Increased levels of TGF-B have also been well documented in smokers (33). These finding suggest that smoking may contribute to the increased amount of TGF-B formation in patients with DM who are already prone to TGF-B mediated nephropathy.

Sauriasair et at. showed that high-sensitivity C-reactive protein (hs-CRP) levels are associated with deterioration of renal function and increased urinary protein output, as a consequence of chronic exposure to oxidative stress induced by smoking (34).

The particulate phase of cigarette smoke possesses over 10^17^ free radicals per gram and vapor phase possesses nearly 10^15^ free radicals per puff (34). Both smoking and DM promote excessive formation of reactive oxygen species (ROS) and diminished capacity of antioxidant molecules to transform ROS (11). Hydroxyl,superoxide and peroxyl which are the main intravascular ROS are formed *via* oxidation of glucose, the sorbitol-aldose reductase pathway, excessive AGE production, NADPH oxidases and mitochondrial oxidative phosphorylation (35).

Research performed on the diabetic renal milieu has shown that certain cell types within the kidney are prone to intracellular hyperglycemia due to functional receptor differences (35). Mesengial, proximal tubular and glomerular epithelial cells exhibit increased glucose uptake and are therefore more susceptible to ROS formation by excess glycolysis (36).

In physiologic state, the homeostasis between the oxidant and the antioxidant mechanisms in cells provide the ideal electrochemical potential difference for optimal cell signaling (37). In smokers and patients with DM, the redox capacity of cells are diminished and the balance shifts towards the pro-oxidative pathway.

Absent endogenous antioxidant defense mechanisms which lead to impaired cell signaling and tissue degeneration has been reported in previous studies. It was shown in diabetic rats with nicotine exposure that the levels of scavenger enzymes, catalase and superoxide dismutase were inversely proportional with lipid peroxidation byproducts (38). Nicotine also inhibits glutathione reductase directly, which results in inadequate reduction of glutathione disulphide (39). Fukami et al. demonstrated that administration of N-acetylcysteine, the precursor of glutathione, reduced mesangial cell hypertrophy and fibronectin synthesis mediated by ROS in patient with DM (40).

Due to chronic hypoxic states, endothelin-1 and vascular endothelial growth factor (VEGF) levels are increased in smokers (6). Raised VEGF levels induce higher mitosis rates of endothelial cells that results in thickened renal arterioles and increased permeability of endothelial monolayers (34). Then intraglomerular capillary pressure increases which subsequently increase the GFR. The development of kidney fibrosis was observed in rats with T2DM who were exposed to tobacco smoke, during a follow-up period of only 4 weeks (41). Levels of serum nitrate, CRP, endothelin-1 and a prominent oxidation byproduct, thiobarburic acid were significantly elevated in this study compared to groups with isolated DM or isolated tobacco exposure (41).

Nagasawa et al. documented that the progression of DKD due to smoking was inversely proportional to the GFR levels (42). It was postulated that the detrimental effect of smoking on higher stages of kidney disease was due to the cumulative oxidative stress that leads to endothelial damage (42). Consequently, the simultaneous effects of smoking and DKD on ROS generation impose a synergistic negative effect on the renal tissue.

## Hyperlipidemia

Atherogenic dyslipidemia, defined as hyperglycemia, high LDL and low high-density lipoprotein (HDL) is by itself an independent risk factor for DKD progression. Insulin resistance creates a relative insulin deficiency that downregulates the enzyme lipoprotein lipase (LPL) which metabolizes triglyceride(TG) molecules (12). Consequently, TG rich very low density lipoprotein (VLDL) levels increase in patients with T2DM (43). In addition to the defective removal of VLDL, VLDL catabolism is further impaired in patients with DM (44). apoC3 expression is suppressed by insulin *via* downregulation of hepatic FOXO1 gene (45).

Higher levels of LDL, VLDL and TG with lower levels of HDL are observed in smokers, indicating the profound effect of tobacco on dyslipidemia. The precise mechanisms of the contribution of nicotine to the pathogenesis of dyslipidemia are not fully established. Notwithstanding, several studies have shown that smoking increases sterol regulatory element binding protein-1 (SREBP-1), that modulates TG and LDL synthesis (46). Increased SREBP-1 levels were reported in a study performed on rats with DKD, stating the role of SREBP-1 mediated lipid deposition, that contributes to tubulointerstitial fibrosis, glomerulosclerosis and albuminuria (47).

The abundance of cholesterol molecules in the diabetic patients and smokers enhance glomerulosclerosis directly by increasing TGF-B levels. Mesangial and tubular cell cultures in a medium with above normal concentrations of LDL have been documented to induce TGF-B expression (48).

Apart from the harmful functional aspect of lipid molecules to the renal vasculature, Narwot et al. suggested that oxidized-LDL may be a biomolecular marker of systemic vascular disease in smokers (49). Oxidized LDL also plays a major role in the signaling pathway of eNOS uncoupling by the displacement of eNOS from the caveole to the cytoplasm, which results in decreased NO production (50). It has been shown in patients with DM that glycated LDL molecules are more prone to oxidation (51). Furthermore, tobacco smoke itself enhances LDL oxidation (52). Yamaguchi et al. demonstrated that oxidized LDL levels rise rapidly after smoking *via* mechanisms that lead to peroxynitrate formation (53). Thus, the combined synergistic effect on LDL oxidation may explain the exacerbation of DKD progression in smokers.

In a study where diabetic nephropathy was induced to rats, adipophilin, an adipocyte differentiation related protein that shows cytoplasmic fat deposition was higher than normal (43). This highlights the significance to recognize dyslipidemia not only as a pro-atherogenic metabolic state but also to acknowledge the causality of glomerulosclerosis.

## Diagnosis of DKD

Presently, even though it lacks specificity and sensitivity, microalbuminuria is a widely used clinical indicator for DKD in patients with DM (54). Latest guidelines recommend checking spot urine albumin/creatinine ratio during each outpatient visit. Screening for patients with T1DM is advised 5 years after diagnosis while screening of patients with T2DM should be done at the time of diagnosis (55).

Currently, diabetic glomerulosclerosis proven by renal biopsy is the gold standard diagnostic test for DKD however, the biopsy rates are low compared to the estimated disease prevalence (4). Apart from being an invasive and therefore risky procedure, it is usually performed on patient with atypical presentation (e.g. deteriorating proteinuria, systemic symptoms, absence of pre-existing microvascular complications) (4). Structural alterations and phenotypic changes can be observed in biopsy specimens 2-8 years before clinically overt DM (55).

Risk stratification of DKD progression based on several biomarkers like the proteomic profile of the urine was investigated in recent studies (4). Papale et al. analyzed the urine protein profiles of T2DM patients and concluded that the prominent predictors of DKD were Ubiquitin and B2- macroglobulin (56). Early diagnosis of DKD by urinary excretion of these biomarkers was proposed as a novel clinical approach (37). In patients with T2DM, circulating levels of Tumor necrosis factor – α (TNF-α) receptors were proven to correlate with DKD progression (55). Al-Rubeaan et al. extensively studied 22 potential biomarkers of DKD and found that the 3 biomarkers (urinary retinol binding protein, urinary transferrin, serum osteopontin) had excellent diagnostic accuracy (57). Urine transferrin/creatinine ratio was correlated to albumin/creatinine ratio in another cohort from Japan (58). Considering that albuminuria is not specific to DKD, definitive biomarkers would be practice changing and would make a substantial impact on preventative medicine. A mass spectrometry method named CKD273 is a point based system based on 273 urinary proteins was used in normoalbuminuric patients with T2DM in the PRIORITY trial (59, 60). Patients with high risk scores determined by the urinary proteomic classifier CKD273 were associated with progression to microalbuminuria (60). Even though some of these mentioned biomarker tests are commercially available, they are expensive methods with variable specificities.

To date, none of these biomarkers are included in major DM guidelines. However, future findings may guide clinicians to individually assess the likelihood of nephropathy in diabetic patients and plan a holistic preventative DM treatment.

## Conclusion

The prevalence of DM is increasing rapidly while implementing measures to decelerate disease progression with medications and preventative strategies are the current mainstay approach. Diabetic nephropathy is a cause of morbidity and mortality while also being an intense burden on the healthcare system. Smoking imposes multi factorial deleterious effect on renal physiology. Smoking is an established risk for DKD, and there is an interplay between hyperglycemia and tobacco exposure that exacerbate renal deterioration *via* mechanism of sustained sympathetic activity, atherosclerosis, oxidative stress and hyperlipidemia. There are overlaps in certain systems where the detrimental effect of DM and smoking combined may results in unforeseen acceleration of kidney dysfunction. This review provides various pathways that explain smoking mediated progression of DKD and highlight the importance of glycemic state and smoking cessation for its prevention. Novel biomarkers that may be implemented to clinical practice in the future that predict risk of DKD progression are also briefly mentioned. More focused investigation regarding these biomarkers and their reflection to preventative healthcare are essential. Prospective studies with long term follow- up periods are needed to determine specific risk groups for DKD progression in smokers.

## Author Contributions

İA contributed to the conceptualization, methodology, supervision, and writing—review and editing process. YG contributed to writing—review and editing, and project administration. Both authors contributed to the article and approved the submitted version.

## Conflict of Interest

The authors declare that the research was conducted in the absence of any commercial or financial relationships that could be construed as a potential conflict of interest.

## Publisher’s Note

All claims expressed in this article are solely those of the authors and do not necessarily represent those of their affiliated organizations, or those of the publisher, the editors and the reviewers. Any product that may be evaluated in this article, or claim that may be made by its manufacturer, is not guaranteed or endorsed by the publisher.
